# Supplementary material for: Ribonucleotide reductase, a novel drug target for gonorrhea
Source: eLife. 2022 Feb 9;11:e67447. doi: 10.7554/eLife.67447 (PMC8865847; doi:10.7554/eLife.67447)
Supplement: Supplementary file 1. — Two isolates gave elevated PTC-847 or PTC-672 MIC values. However, these isolates were sensitive to all other antibiotics. Due to the selectivity of the PTC compounds, these two strains will be genotyped to confirm they are Neisseria species. PTC-compound susceptibility testing was performed in accordance with the Clinical and Laboratory Standards Institute (CLSI) M07-A9 guideline (Clinical and Laboratory Standards Institute, 2012). [file elife-67447-supp1.docx]

| **Antibiotic / Compound** | **MIC range**  **(µg/mL)** | | **Value for decreased susceptibility (µg/mL)** | **Value for elevated MICs (µg/mL)** | **Isolates with elevated MICs** |
| --- | --- | --- | --- | --- | --- |
| Azithromycin | ≤0.03 to >256 | >8500-fold | ≥2 | Na | 9 |
| Cefixime | ≤0.002 to 4 | >2000-fold | ≥0.5 | ≥0.25 | 16 |
| Ceftriaxone | ≤0.002 to 1 | >500-fold | ≥0.5 | ≥0.12 | 5 |
| Ciprofloxacin | ≤0.03 to >16 | >500-fold | ≥0.12 | Na | 71 |
| Tetracycline | <0.5 to >16 | >32-fold | ≥2 | Na | 179 |
| PTC-847 | 0.03 to >1 | >32-fold | - | >1 | 2 |
| PTC-672 | 0.015 to 0.25 | 16-fold | - | - | 2 |
