# Supplementary material for: Ribonucleotide reductase, a novel drug target for gonorrhea
Source: eLife. 2022 Feb 9;11:e67447. doi: 10.7554/eLife.67447 (PMC8865847; doi:10.7554/eLife.67447)
Supplement: Supplementary file 5. — By plating at high cell density on agar plates containing PTC-847 at 4, 8, 16 and 32-fold MIC, Ng 13477 exhibited a spontaneous or acquired frequency of resistance to PTC-847 on the order of 10–8. The colonies obtained were passaged multiple times on PTC-847-containing plates to obtain a stable PTC-847-resistant strain (PTC-847R). [file elife-67447-supp5.docx]

| **Strain** | **MIC fold** | **Resistance Frequency** | | |
| --- | --- | --- | --- | --- |
|  |  | **PTC-847** | **Ciprofloxacin** | **Ceftriaxone** |
| *Ng* 13477 | 32X | 5.6 x 10^-9^ | ≤8 x 10^-9^ | ─ |
|  | 16X | 5.6 x 10^-9^ | ≤8 x 10^-9^ | ─ |
|  | 8X | 1.1 x 10^-8^ | ≤8 x 10^-9^ | ≤1.9 x 10^-9^ |
|  | 4X | 3.6 x 10^-8^ | 1.2 x 10^-8^ | 7.7 x 10^-9^ |
