# Supplementary material for: Ribonucleotide reductase, a novel drug target for gonorrhea
Source: eLife. 2022 Feb 9;11:e67447. doi: 10.7554/eLife.67447 (PMC8865847; doi:10.7554/eLife.67447)
Supplement: Supplementary file 6. — Susceptibility of the WT Ng 13477 strain and the PTC-847R strain were measured for a wide variety of antibiotics having different modes of action. MICs were determined in accordance with the CLSI M07-A9 guideline (Clinical and Laboratory Standards Institute, 2012). The PTC-847R strain was equally sensitive to all classes of antibiotics as the susceptible WT Ng 13477 strain, except for resistance to PTC-847. The PTC-847 MIC for WT Ng 13477 was 0.05 µg/mL compared to 15.6 µg/mL for the PTC-847R strain. [file elife-67447-supp6.docx]

| Inhibitor  type | Class | Compound / Antibiotic | *Ng* MIC (µg/mL) | |
| --- | --- | --- | --- | --- |
|  |  |  | Wild-type | PTC-847^R^ |
| DNA | novel | PTC-847 | 0.05 | 15.6 |
|  | fluoroquinolones | ciprofloxacin | 0.003 | 0.003 |
|  |  | moxifloxacin | 0.003 | 0.003 |
|  |  | enrofloxacin | 0.004 | 0.004 |
|  |  | delafloxacin | 0.05 | 0.05 |
| Protein | Oxazolidinone | linezolid | 2 | 2 |
|  | aminoglycosides | gentamicin | 2 | 2 |
|  |  | kanamycin | 6.2 | 6.2 |
|  | Macrolides | solithromycin | 0.1 | 0.05 |
|  |  | erythromycin | 0.2 | 0.2 |
|  | Tetracyclines | tetracycline | 1 | 1 |
|  |  | tigecycline | 0.4 | 0.4 |
| RNA | Rifamycin | rifampicin | 0.1 | 0.1 |
| Cell wall | beta-lactam | ampicillin | 0.06 | 0.06 |
|  | cephalosporins | cefepime | 0.002 | 0.002 |
|  |  | ceftriaxone | 0.0002 | 0.0004 |
