# Supplementary material for: Ribonucleotide reductase, a novel drug target for gonorrhea
Source: eLife. 2022 Feb 9;11:e67447. doi: 10.7554/eLife.67447 (PMC8865847; doi:10.7554/eLife.67447)
Supplement: Supplementary file 7. — The susceptible WT Ng 13477 strain was grown to log phase and treated with DMSO or PTC-847 at 1 X MIC for 1 h. Nucleotides were extracted in acidified acetonitrile/H2O (65:35) followed by centrifugation. The supernatant was lyophilized and subjected to LC/MS. 13C9,15N3─CTP was added for LC/MS analysis. Peak areas of NTP and dNTPs were normalized to the peak area of 13C9,15N3─CTP. [file elife-67447-supp7.docx]

| NTP/dNTP Ratio | Treatment | | Fold change |
| --- | --- | --- | --- |
|  | DMSO | PTC-847  (1X MIC) |  |
| CTP/dCTP | 10.91 | 33.79 | 3.1 |
| UTP/dTTP | 7.63 | 24.12 | 3.2 |
| ATP/dATP | 17.4 | 44.2 | 2.5 |
| GTP/dGTP | 19.9 | 24.9 | 1.3 |
