# Supplementary material for: Ribonucleotide reductase, a novel drug target for gonorrhea
Source: eLife. 2022 Feb 9;11:e67447. doi: 10.7554/eLife.67447 (PMC8865847; doi:10.7554/eLife.67447)
Supplement: Supplementary file 10. — The panel was constructed to test for inhibition of normally occurring intestinal organisms (Thursby and Juge, 2017). Susceptibility testing was performed in accordance with the Clinical and Laboratory Standards Institute (CLSI) M07-A9 guideline (Clinical and Laboratory Standards Institute, 2012). PTC-672 was tested against a more extensive panel of organisms. [file elife-67447-supp10.docx]

|  |  | **MIC (µg/mL)** | | |
| --- | --- | --- | --- | --- |
| **Organism** | **ATCC #** | **PTC-672** | **PTC-847** | **Solithromycin** |
| *Bacteroides fragilis* | 25285 | >32 | 32 | 1 |
| *Bacteroides ovatus* | 8483 | >32 |  | 1 |
| *Bifidobacterium longum* | 15707 | >32 | 16 | ≤ 0.03 |
| *Bifidobacterium bifidum* | 15696 | >32 | 32 | ≤ 0.03 |
| *Clostridium difficile* | 700057 | 32 | 32 | 0.06 |
| *Clostridium perfringens* | 13124 | >32 |  | ≤ 0.03 |
| *Eubacterium lentum* | 43055 | >32 |  | 0.06 |
| *Lactobacillus acidophilus* | 4356 | >32 |  | ≤ 0.03 |
| *Lactobacillus casei* | 393 | >32 |  | ≤ 0.03 |
| *Lactobacillus crispatus* | 33820 | >32 |  | 0.06 |
| *Lactobacillus jensenii* | 25258 | >32 |  | ≤ 0.03 |
| *Lactobacillus gasseri* | 33323 | >32 |  | ≤ 0.03 |
| *Lactobacillus vaginalis* | 49540 | >32 |  | ≤ 0.03 |
| *Peptostreptococcus micros* | Unknown | >32 |  | 0.06 |
| *Peptostreptococcus magnus* | 14596 | >32 |  | 0.03 |
| *Eikenella corrodens* | 43278 | >32 |  | 0.5 |
| *Fusobacterium nucleatum* | 25586 | >32 |  | 0.5 |
| *Prevotella bivia* | 29303 | >32 |  | 0.06 |
| *Prevotella oralis* | 33269 | >32 |  | ≤ 0.03 |
| *Neisseria meningitidis* | 13090 | 0.06 |  | 0.06 |
