# Supplementary material for: Ribonucleotide reductase, a novel drug target for gonorrhea
Source: eLife. 2022 Feb 9;11:e67447. doi: 10.7554/eLife.67447 (PMC8865847; doi:10.7554/eLife.67447)
Supplement: Supplementary file 11. — Two commensal Neisseria ‘type strains’ swabbed from the oropharynx of healthy volunteers (†Berger, 1971 and ‡Riou and Guibourdenche, 1987) were tested for susceptibility to inhibition in accordance with the Clinical and Laboratory Standards Institute (CLSI) M07-A9 guideline (Clinical and Laboratory Standards Institute, 2012). [file elife-67447-supp11.docx]

| **Compound** | MIC (µg/mL) | | |
| --- | --- | --- | --- |
|  | *N. mucosa* ATCC25996^†^ | *N. polysaccharea* 43768^‡^ | *Ng* 13477 |
| Broad spectrum | 0.78 | 0.39 | 0.098 |
| AZD0914 | 0.78 | 0.39 | 0.049 |
| Ciprofloxacin | 0.008 | 0.008 | 0.004 |
| PTC-672 | 1.56 | 0.39 | 0.05 |
