# Supplementary material for: Ribonucleotide reductase, a novel drug target for gonorrhea
Source: eLife. 2022 Feb 9;11:e67447. doi: 10.7554/eLife.67447 (PMC8865847; doi:10.7554/eLife.67447)
Supplement: Supplementary file 12. [file elife-67447-supp12.docx]

| **Species** | **Class Ia** | **Class Ib** | **Class Ic** | **Class II** | **Class III** |
| --- | --- | --- | --- | --- | --- |
| *N. gonorrhoeae* | Yes |  |  |  |  |
| *N. meningitidis* | Yes |  |  |  |  |
| *E. coli* | Yes | Yes |  |  | Yes |
| *K. pneumoniae* | Yes | Yes |  |  | Yes |
| *B. subtilis* |  | Yes |  |  |  |
| *P. aeruginosa* | Yes |  |  | Yes | Yes |
| *C. trachomatis* |  |  | Yes |  |  |
| *B. fragilis* | Yes |  |  | Yes | Yes |
| *C. difficile* |  | Yes |  | Yes | Yes |
| *L. gasseri* |  | Yes |  | Yes | Yes |
| *F. nucleatum* | Yes |  |  |  | Yes |
