# Supplementary material for: Ribonucleotide reductase, a novel drug target for gonorrhea
Source: eLife. 2022 Feb 9;11:e67447. doi: 10.7554/eLife.67447 (PMC8865847; doi:10.7554/eLife.67447)
Supplement: Supplementary file 8 [file elife-67447-supp2.docx]

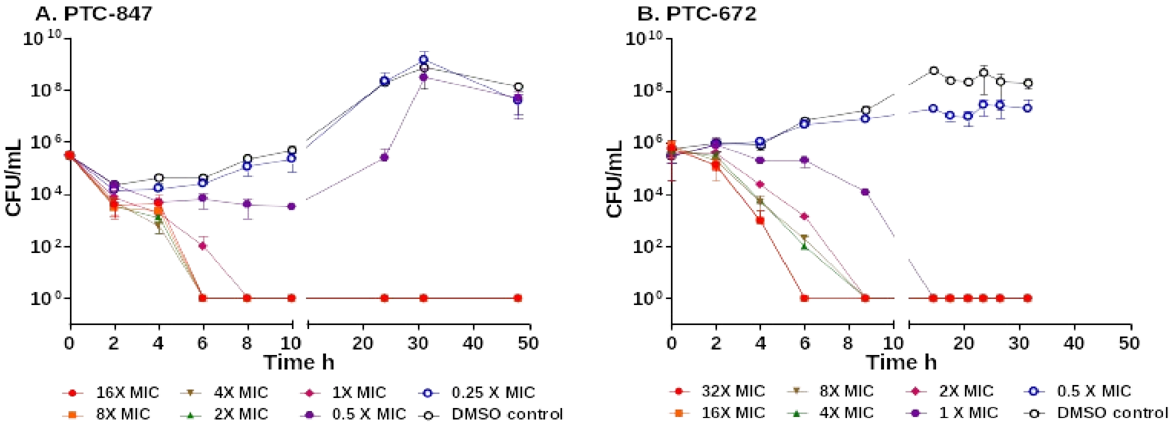
A. PTC-847
B. PTC-672
CFU/mL
Time h
16X MIC
8X MIC
4X MIC
2X MIC
1X MIC
0.5 X MIC
0.25 X MIC
DMSO control
32X MIC
16X MIC
8X MIC
4X MIC
2X MIC
1 X MIC
0.5 X MIC
DMSO control
