# Supplementary material for: Ribonucleotide reductase, a novel drug target for gonorrhea
Source: eLife. 2022 Feb 9;11:e67447. doi: 10.7554/eLife.67447 (PMC8865847; doi:10.7554/eLife.67447)
Supplement: Supplementary file 9 [file elife-67447-supp3.docx]

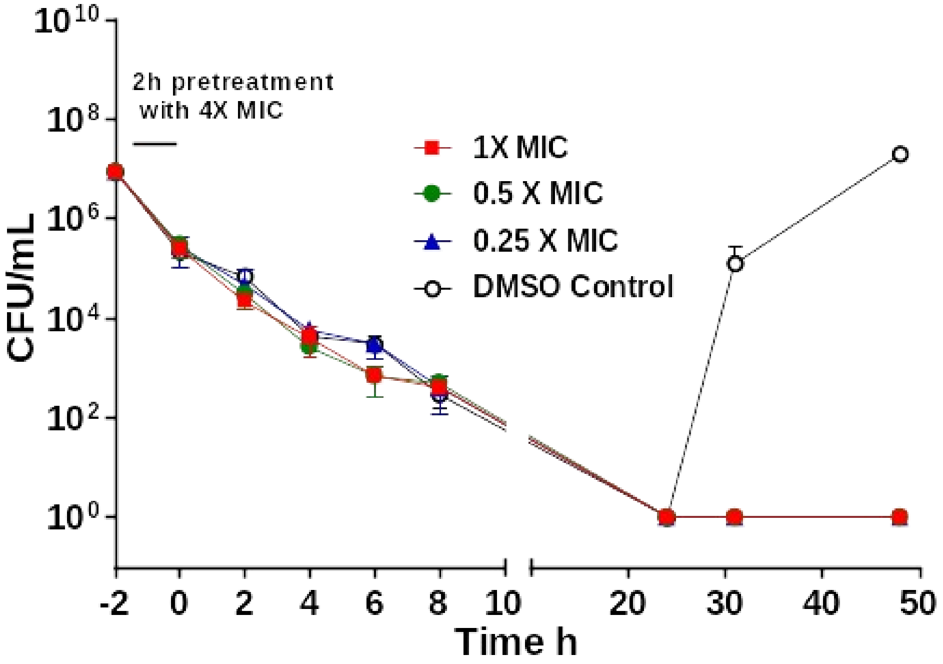

10^{10}
10^{8}
10^{6}
10^{4}
10^{2}
10^{0}
CFU/mL
2h pretreatment
with 4X MIC
1X MIC
0.5 X MIC
0.25 X MIC
DMSO Control
-2
0
2
4
6
8
10
20
30
40
50
Time h
