# Supplementary material for: Ribonucleotide reductase, a novel drug target for gonorrhea
Source: eLife. 2022 Feb 9;11:e67447. doi: 10.7554/eLife.67447 (PMC8865847; doi:10.7554/eLife.67447)
Supplement: Supplementary file 10 [file elife-67447-supp4.docx]

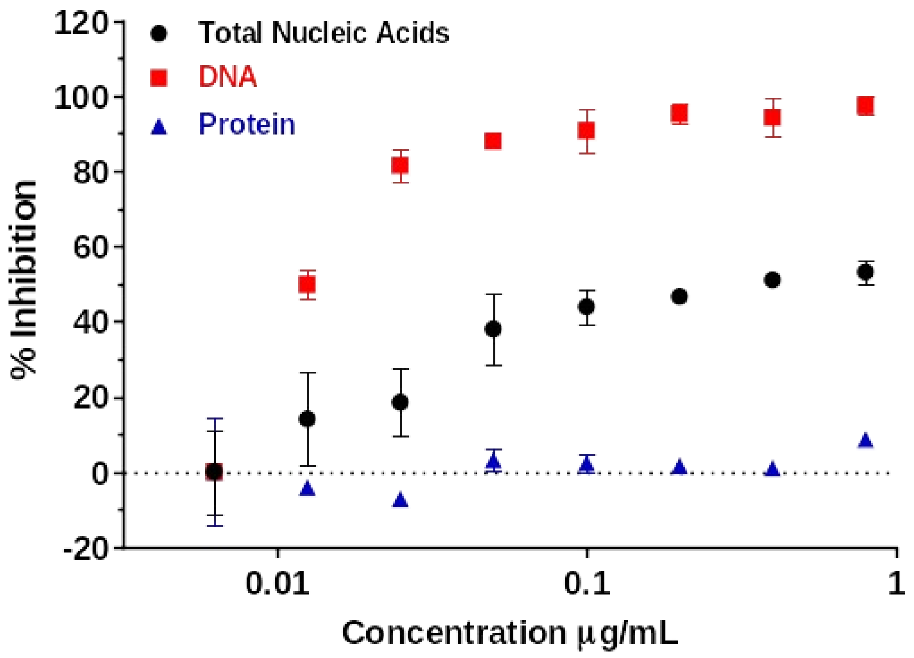

Total Nucleic Acids
DNA
Protein
% Inhibition
120
100
80
60
40
20
0
-20
0.01
0.1
1
Concentration µg/mL
