# Supplementary material for: Ribonucleotide reductase, a novel drug target for gonorrhea
Source: eLife. 2022 Feb 9;11:e67447. doi: 10.7554/eLife.67447 (PMC8865847; doi:10.7554/eLife.67447)
Supplement: Supplementary file 11 [file elife-67447-supp8.docx]

A

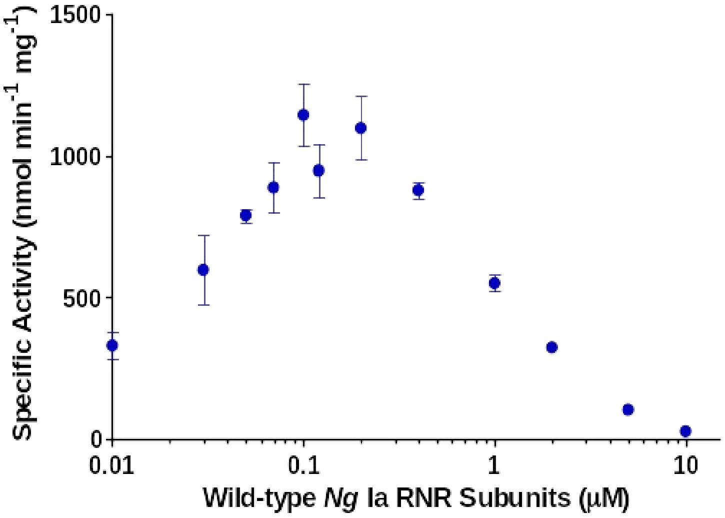

Specific Activity (nmol min-1 mg-1)
1500
1000
500
0
0.01
0.1
1
10
Wild-type Ng Ia RNR Subunits (µM)

B

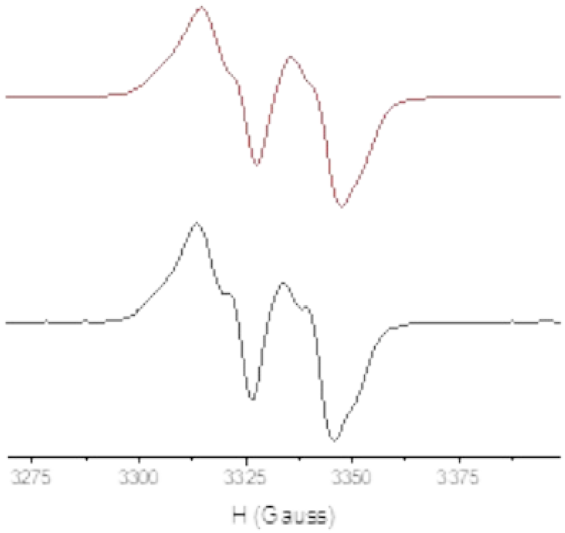

3275
3300
3325
3350
3375
H (Gauss)
