# Supplementary material for: Ribonucleotide reductase, a novel drug target for gonorrhea
Source: eLife. 2022 Feb 9;11:e67447. doi: 10.7554/eLife.67447 (PMC8865847; doi:10.7554/eLife.67447)
Supplement: Supplementary file 12 [file elife-67447-supp9.docx]

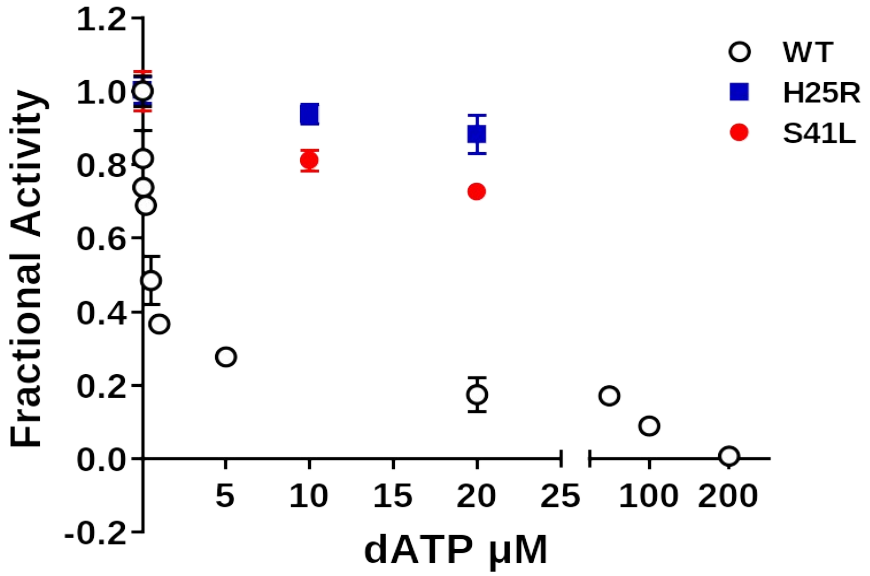

Fractional Activity
1.2
1.0
0.8
0.6
0.4
0.2
0.0
-0.2
5
10
15
20
25
100
200
dATP µM
WT
H25R
S41L
